# Challenges in Managing Newly Diagnosed Granulomatosis With Polyangiitis and Concurrent Respiratory Infections: A Retrospective Case Series

**DOI:** 10.7759/cureus.66412

**Published:** 2024-08-07

**Authors:** Jeevanandham Anandan, Krishnarajasekhar R Ottilingam

**Affiliations:** 1 Respiratory Medicine, Saveetha Medical College and Hospitals, Saveetha Institute of Medical and Technical Sciences, Saveetha University, Chennai, IND

**Keywords:** bronchoalveolar lavage (bal), customized patient care, respiratory tract infection, multidisciplinary discussion, granulomatosis with polyangiitis (gpa), pneumonia, tuberculosis, immunosuppressive treatment, antineutrophil cytoplasmic antibody (anca), necrotizing granulomatous inflammation

## Abstract

Introduction: Granulomatosis with polyangiitis (GPA), formerly termed Wegener's granulomatosis, is an autoimmune disease marked by necrotizing granulomatous inflammation and vasculitis affecting small-sized vessels. It commonly impacts the renal and respiratory systems.

Materials and methods: This retrospective case series sampling conducted in a tertiary care hospital between May 2023 and April 2024 examined six newly diagnosed GPA patients who were proteinase 3 cytoplasmic-antinuclear cytoplasmic antibody (PR3 c-ANCA) positive and had concurrent respiratory infections. None of them had any prior immunosuppressive conditions. The age range was 18-47 years with a mean of 35.0 (standard deviation: 11.83). All the patients had pneumonia (N=6, 100%). Out of all, five had bacterial pneumonia (N=5, 83.3%) and one had tuberculous pneumonia (N=1, 16.7%). A high level of PR3 c-ANCA (>150 RU/mL) was noted in four patients (N=4, 66.7%). Common symptoms included dry cough (N=5, 83.3%), loss of weight and appetite (N=2, 33.3%), and fever (N=2, 33.3%). Three patients had otitis media and/or nasal polyposis (N=3, 50%). Two patients (N=2, 33.3%) with life-threatening organ dysfunction were given concurrent antibiotics and steroids; the antibiotics were later modified based on culture and sensitivity results. One of these patients received antituberculosis therapy as *Mycobacterium tuberculosis* (MTB) was detected after 27 days of incubation in mycobacterial growth indicator tube broth. The remaining four patients (N=4, 66.7%) received antibiotics initially for 5-7 days until clinical resolution of pneumonia. Ultimately, they all showed clinical and radiological resolution (N=6, 100%) within 3-6 months of treatment.

Results: The patients exhibited constitutional symptoms such as fever and weight loss; lower airway disease symptoms including dry cough and hemoptysis; nasal and ear disease symptoms like epistaxis, ear pain, and ear discharge; and a renal disease symptom, hematuria. Computed tomography of the thorax revealed bilateral consolidations, most of which were cavitating. Bronchoalveolar lavage cultures grew *Escherichia coli*, *Burkholderia cepacia*, *Pseudomonas aeruginosa*, *Klebsiella pneumoniae,* and MTB, whereas pus swab cultures from otitis media grew *Pseudomonas aeruginosa*, *Staphylococcus aureus, *and coagulase-negative staphylococci.

Discussion: This study highlights the therapeutic challenges of GPA complicated by concurrent infections. Patients exhibited typical GPA signs, confirmed by PR3 c-ANCA levels. Concurrent infections require cautious antibiotic treatment before starting immunosuppressive therapy, except in life-threatening organ dysfunction. A unique case presented with both tuberculosis and GPA. Tailored treatment regimens combining antibiotics and immunosuppressives, including corticosteroids, methotrexate, and rituximab, resulted in clinical and radiological improvement in all the patients within 3-6 months. The addition of co-trimoxazole reduced the incidence of non-severe GPA relapses.

Conclusion: Tailored treatment plans addressing both infectious and autoimmune aspects are essential for optimal care in GPA complicated by concurrent infections. This study highlights the need for a multidisciplinary approach involving pulmonologist, rheumatologist, microbiologist, and pathologist in the diagnosis and treatment of GPA, emphasizing the importance of individualized treatment plans tailored to the specific clinical scenario.

## Introduction

Granulomatosis with polyangiitis (GPA), previously called as called Wegener's granulomatosis, is an autoimmune disease that affects small-to-medium-sized arteries and is characterized by necrotizing granulomatous inflammation and vasculitis [1]. The clinical presentation of GPA can vary widely, ranging from mild symptoms to severe, multiorgan involvement/failure. It usually affects the kidneys and lungs and frequently manifests as a cluster of non-specific symptoms like fever, persistent cough, and systemic signs like weight loss. The disease is often associated with the presence of antineutrophil cytoplasmic antibodies (ANCA), specifically proteinase 3 (PR3)-ANCA, which is commonly detected in the serum of affected individuals. Early and accurate diagnosis is critical, as the disease can rapidly progress to organ failure [2].

However, the diagnosis of GPA can be challenging due to its overlap with infectious diseases, which can both mimic and coexist with the autoimmune condition [3]. It is imperative to promptly identify and address any coexisting infections prior to commencing immunosuppressive therapy in order to prevent the infection from worsening and affecting patient outcomes [4]. The presence of concurrent infections at the time of GPA diagnosis poses diagnostic and therapeutic challenges, as it can complicate the clinical picture and delay appropriate treatment. Infections in GPA patients can mimic disease manifestations, making it difficult to distinguish between infectious and autoimmune processes.

This study provides novel insights and contributes to the existing literature on this topic. This case series describes six patients newly diagnosed with GPA, all of them presented with concurrent respiratory infections. None of the patients had comorbidities such as human immunodeficiency virus (HIV) infection or diabetes, which are commonly associated with increased susceptibility to infections. The clinical presentation, diagnostic challenges, treatment strategies, and outcomes of these patients are discussed to provide insights into the complexities of managing newly diagnosed GPA patients with concurrent infections. This case series also highlights the importance of a multidisciplinary approach and the need for individualized treatment plans to achieve optimal outcomes. We provided a simplified flow chart for managing patients of newly diagnosed GPA with lung consolidation.

## Materials and methods

This retrospective case series analyzed the data of GPA patients who were treated in the Department of Respiratory Medicine from May 2023 to April 2024. We included six patients of newly diagnosed GPA, proteinase-3 cytoplasmic antinuclear cytoplasmic antibody (PR3 c-ANCA), without any comorbidities (HIV infection or diabetes) who had concurrent lower and/or upper respiratory infections. Demographic data like age, gender, and occupation, and clinical data like symptoms, vitals, ear, nose, and eye examination were noted. All the patients had chest X-rays and computed tomography (CT) of thorax during admission and follow-up. Bronchoscopy was done for all and bronchoalveolar lavage (BAL) sample was sent for investigations like Gram stain, bacterial culture (blood/chocolate agar), acid-fast bacilli smear, Gene Xpert, mycobacterial growth indicator tube (MGIT) culture, potassium hydroxide (KOH) mount, and fungal culture. Investigations like proteinase-3 cytoplasmic antinuclear cytoplasmic antibody (PR3 c-ANCA), white blood cell (WBC) count, platelet count, urine routine, urea, creatinine, urine protein creatinine ratio (PCR) and liver function tests (LFT) were included. The patients who had nasal polyposis underwent nasal polyp biopsy, those who had deranged renal function underwent renal biopsy, and those who had lung nodules underwent CT-guided lung biopsy. Their histopathologic examination findings were noted. The outcome was assessed using the resolution of symptoms and chest X-rays during follow-up at the first, third, and sixth months. Finally, patient's treatment and outcome were noted. Table 1 provides the summary of clinical data and baseline investigations and Table 2 provides bronchoscopy, histopathology, treatment, and follow-up details of all the patients.

**Table 1:** The demography, clinical findings, and investigations of all six GPA patients. RBS: random blood sugar; HIV: human immunodeficiency virus; WBC: white blood cells; RBC: red blood cells; RFT: renal function test; LFT: liver function test; CT: computed tomography; c-ANCA: cytoplasmic antineutrophil cytoplasmic antibody; ASOM: acute suppurative otitis media; CSOM: chronic suppurative otitis media; CoNS: coagulase-negative staphylococci; GPA: granulomatosis with polyangiitis

Features	Patient A	Patient B	Patient C	Patient D	Patient E	Patient F
Age (years)	18	47	44	45	20	36
Gender	Female	Female	Female	Male	Male	Female
Occupation	Student	Housewife	Housewife	Teacher	Mason	Construction worker
Symptoms	Fever for 30 days, dry cough for 25 days, and right ear pain for 20 days	Redness and watering of eyes for 3 months, dry cough for 2 months	Dry cough for 20 days, nasal blockade for 15 days, loss of weight and loss of appetite	Hemoptysis for 45 days, loss of weight and appetite for 1 month	Epistaxis and nasal stuffiness for 4 months. Dry cough for 2 months	Right ear discharge for 2 months, fever for 15 days, dry cough for 15 days, hematuria for 10 days
Vitals	Stable	Stable	Stable	Stable	Stable	Stable
RBS (normal <200 mg/dL)	88	170	112	121	132	78
Diabetes	Absent	Absent	Absent	Absent	Absent	Absent
HIV	Negative	Negative	Negative	Negative	Negative	Negative
Immunosuppressants	Naive	Naive	Naive	Naive	Naive	Naive
WBC count (reference range: 4,000-11,000 cells/mm^3^)	15,000	9,730	13,310	11,500	8,500	16,010
Platelet count (reference range: 1,50,000-4,50,000 cells/mm^3^)	7,44,000	5,79,000	3,02,000	4,50,000	3,50,000	2,50,000
Urine routine	Normal	Microscopic hematuria with RBC casts +	Normal	Normal	Normal	Gross hematuria with RBC casts +
Urea (reference range: 20-40 mg/dL)	Normal	58	Normal	Normal	Normal	65
Creatinine (normal <1.0 mg/dL)	Normal	1.7	Normal	Normal	Normal	2.5
Urine PCR (normal <0.2 mg/g)	Normal	1.52	Normal	Normal	Normal	2.61
LFT	Normal	Normal	Normal	Normal	Normal	Normal
Chest X-ray	Bilateral rounded non-homogenous opacities in all zones	Right lower zone non-homogenous opacities	Right upper and left lower zones rounded non-homogenous opacities	Right lower zone and left upper zone non-homogenous opacities	Right mid zone rounded opacity, left pleural effusion	Right mid and lower zones non-homogenous opacities
CT thorax	Bilateral multilobar consolidation and cavitating nodules	Right lower lobe consolidation and left lower lobe cavitating consolidation	Bilateral multilobar consolidation and cavitating nodules	Left upper lobe and right lower lobe consolidation	Not done	Right middle lobe consolidation
Ear examination	Left ASOM: treated with ciprofloxacin ear drops	Normal	Right ASOM: methicillin-resistant CoNS: treated with gentamycin ear drops	Normal	Normal	Right CSOM: treated with ciprofloxacin ear drops
Nose examination	Left nasal polyposis	Normal	Right nasal polyposis	Normal	Left nasal polyposis	Normal
Eye examination	Normal	Bilateral eye scleritis and scleral necrosis - treated with moxifloxacin + dexamethasone eye drops	Normal	Normal	Normal	Normal
Proteinase 3 c-ANCA (RU/mL) - reference range: <20: negative, ≥20: positive	212.3	>200	92.6	121	180	232.8

**Table 2:** Bronchoscopy, histopathology, treatment, and follow-up details of all the patients. BAL: bronchoalveolar lavage; CBNAAT: cartridge-based nucleic acid amplification test; MTB: mycobacterium tuberculosis; ATT: antitubercular therapy; MGIT: mycobacterial growth indicator tube; HPE: histopathological examination; ANCA: antineutrophil cytoplasmic antibody

Features	Patient A	Patient B	Patient C	Patient D	Patient E	Patient F
Bronchoscopy	No endobronchial pathology	No endobronchial pathology	No endobronchial pathology	Right intermediate bronchus mucosal ulcerations with whitish membranes and erythema with bleeding spots	No endobronchial pathology	No endobronchial pathology
BAL bacterial culture and sensitivity	Escherichia coli sensitive to/treated with piperacillin + tazobactam	Normal upper respiratory tract flora	Burkholderia cepacia sensitive to/treated with meropenem	Pseudomonas aeruginosa sensitive to/treated with ceftazidime	Klebsiella pneumonia sensitive to/treated with meropenem	Pseudomonas aeruginosa sensitive to/treated with ceftazidime
BAL Gene Xpert MTB/RIF	MTB not detected	MTB not detected	MTB not detected	MTB not detected	MTB not detected	MTB not detected
BAL MGIT culture	No growth	Grew MTB after 27 days of incubation, treated with ATT for 6 months	Not done	Not done	No growth	No growth
BAL fungus culture	No growth	No growth	Not done	Not done	No growth	No growth
HPE	Nasal polyp biopsy: features of necrotizing granulomatous inflammation with vasculitis	Kidney biopsy: ANCA-associated crescentic glomerulonephritis pattern of injury - late crescentic and necrotizing glomerulonephritis. Given the prominent interstitial histiocytic infiltration and the presence of Langhans-type giant cells, TB culture and CBNAAT are advised to exclude the possibility of coexisting tuberculosis	Lung biopsy: necrotizing granulomatous inflammation with vasculitis	Endobronchial mucosal biopsy: necrotizing granulomatous inflammation with vasculitis	Nasal polyp biopsy: necrotizing granulomatous inflammation with vasculitis	Kidney biopsy: ANCA-associated crescentic glomerulonephritis pattern of injury - crescentic and necrotizing glomerulonephritis
Organ/life-threatening disease	No	Yes	No	No	No	Yes
Immunosuppressive treatment	Tab. prednisolone 30 mg OD for 2 weeks (then tapered)	Inj. methylprednisolone 1 g OD and 500 mg OD for next 2 days and Tab. prednisolone 40 mg 1-0-0 x 2 weeks, (then tapered)	Tab. prednisolone 45 mg 1-0-0 x 2 weeks (then tapered), Tab. methotrexate 10 mg/week on Fridays, and folate 5 mg/week on Saturdays	Tab. prednisolone 70 mg 1-0-0 x 2 weeks (then tapered) and Tab. cyclophosphamide 150 mg OD	Tab. prednisolone 50 mg OD for 2 weeks (then tapered), Tab. methotrexate 12.5 mg/week, and folate 5 mg/week	Inj. methylprednisolone 1 g OD for 3 days, Tab. prednisolone 35 mg 1-0-0 x 2 weeks (then tapered), and Inj. rituximab was given for remission induction and maintenance
Outcome: X-ray after 3-6 months of treatment	Showed resolution after 4 months	Near complete resolution after 6 months	Near complete resolution after 4 months	Near complete resolution in a month	Near complete resolution in 3 months	Complete resolution in 3 months

Statistical analysis

Continuous data were represented either as mean and standard deviation or median with minimum and maximum values based on normalcy, and categorical data were represented as frequencies and percentages. IBM SPSS Statistics (Armonk, NY: IBM Corp.) version 25.0 was utilized for doing statistical computations.

Data analysis

The study included participants aged 18-47 years, with a mean age of 35.0 years (standard deviation: 11.83). The gender distribution comprised two males (33.3%) and four females (66.7%). At presentation, common symptoms included dry cough in 83.3% (N=5) of the cases, fever in 33.3% (N=2), and loss of weight and appetite in 33.3% (N=2). Other symptoms observed were hemoptysis, epistaxis, nasal discharge, and ear discharge, each affecting 16.7% (N=1) of the patients.

In terms of respiratory infections, all patients had lower respiratory infections (pneumonia) (N=6, 100%), and 66.7% (N=3) had upper respiratory infections (otitis media). The majority experienced bacterial pneumonia (N=5, 83.3%), while one patient had pulmonary tuberculosis (N=1, 16.7%). In terms of ENT manifestations, four patients (66.7%) had ear and nasal diseases, with three having nasal polyposis (N=3, 50%), two with acute suppurative otitis media (N=2, 33.3%), and one with chronic suppurative otitis media (N=1, 16.7%).

Regarding laboratory findings, the median white blood cell (WBC) count was 12,405 cells/mm³, ranging from 8,500 to 16,010 cells/mm³, while the median platelet count was 400,000 cells/mm³, with values ranging from 250,000 to 744,000 cells/mm³. Renal function tests showed that two patients (N=2, 33.3%) had elevated urea and creatinine levels with RBC casts and hematuria, leading to renal biopsies.

PR3 c-ANCA levels were elevated (≥150 U/mL) in four patients (N=4, 66.7%). The median c-ANCA level was 190 U/mL, with a range of 92.6-232.8 U/mL. All patients received corticosteroids as part of their treatment regimen. Concurrent antibiotics and steroids were given to two patients (N=2, 33.3%) as they had life-threatening organ dysfunction. Later, the antibiotic was modified based on culture and sensitivity. One among the former was given antitubercular therapy (ATT) as the MGIT grew *Mycobacterium tuberculosis* (MTB) after 27 days of incubation in MGIT culture. All others who had non-organ/life-threatening disease (N=4, 66.7%) received antibiotics for an initial five to seven days until there was clinical resolution of pneumonia.

All six patients achieved clinical and radiological resolution within three to six months of treatment, except for one patient who showed resolution within a month.

## Results

Case details

Patient A

**Figure 1:**
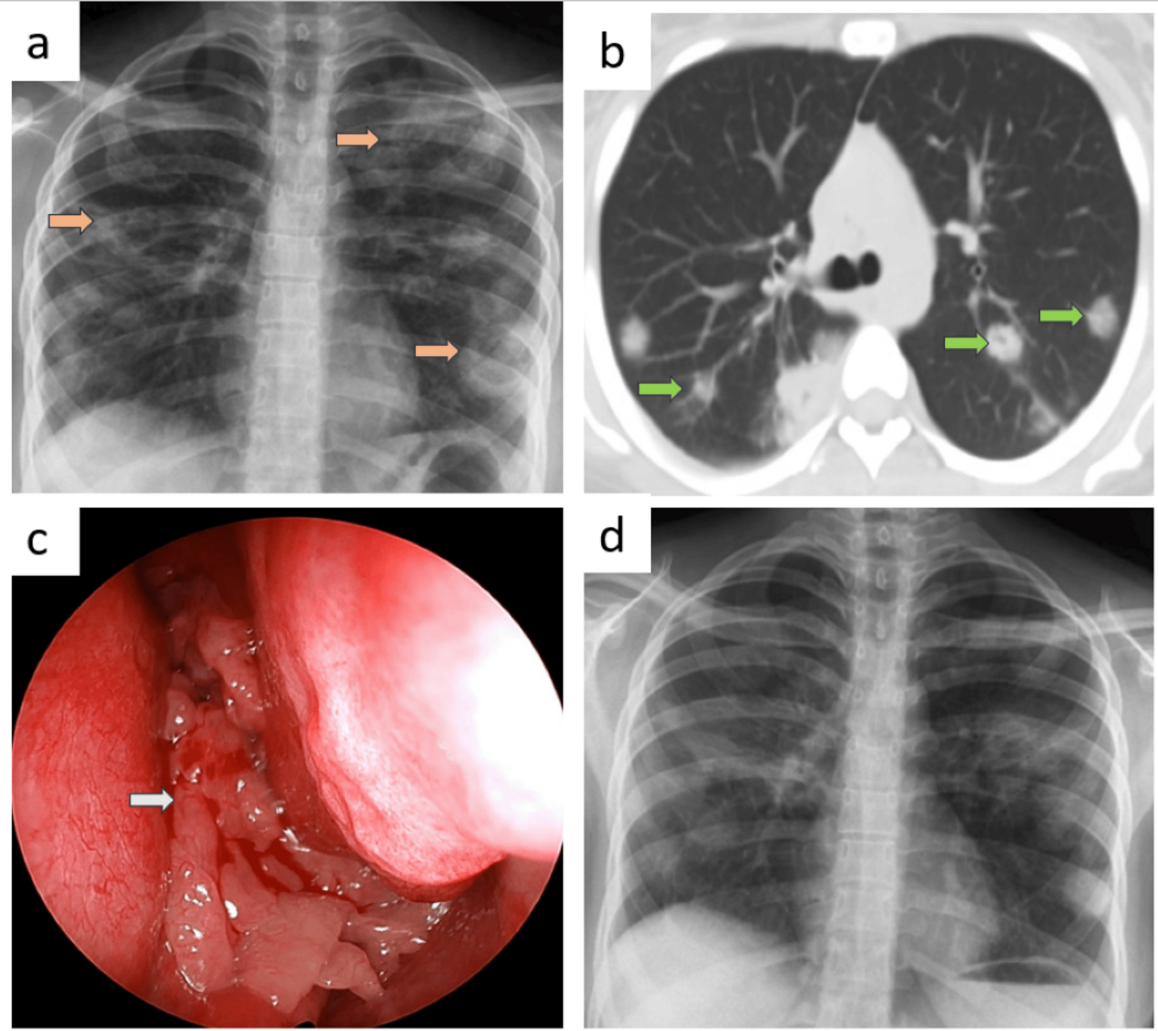
Radiology and anterior rhinoscopy images of patient A at diagnosis and during follow-up. (a) Chest X-ray imaging indicated bilateral rounded non-homogenous opacities across all lung zones marked by brown arrows. (b) Computed tomography (CT) revealed bilateral multilobar lung consolidation and cavitating nodules marked by green arrows. (c) Anterior rhinoscopy examination showed multiple glistening cluster of grapes-like lesions suggestive of left nasal polyposis marked by a white arrow. (d) Follow-up chest radiography after four months of treatment showed resolution of lesions.

A 17-year-old female student presented with a fever lasting 30 days, accompanied by a dry cough for 25 days and right ear pain for 20 days. Her vital signs, including pulse rate, blood pressure, oxygen saturation, and respiratory rate, were stable upon examination. Laboratory investigations revealed a random blood sugar (RBS) level of 88 mg/dL, elevated white blood cell (WBC) count at 15,000 cells/mm³, and an increased platelet count of 744,000 cells/mm³. Routine urine analysis and renal function tests were within normal limits, as were liver function tests (LFT). Chest X-ray imaging indicated bilateral rounded non-homogenous opacities across all lung zones (Figure 1, panel a), while further computed tomography (CT) radiological assessment identified bilateral multilobar lung consolidation and cavitating nodules (Figure 1, panel b). A nasal examination revealed a left nasal polyp (Figure 1, panel c). Notably, PR3 c-ANCA level was significantly elevated at 212.3 RU/mL, well above the positive threshold of 20 RU/mL, confirming the diagnosis of GPA.

Bronchoalveolar lavage (BAL) bacterial culture grew *Escherichia coli*, which was (sensitive to) treated with piperacillin and tazobactam. BAL acid-fast bacilli (AFB) smear showed no AFB, Gene Xpert hadn’t detected *Mycobacterium tuberculosis* (MTB), and fungal culture showed no growth. The patient also had left acute suppurative otitis media (ASOM), which grew *Pseudomonas aeruginosa* in culture and hence treated with ciprofloxacin ear drops. Histopathological examination (HPE) of a nasal polyp biopsy revealed features of necrotizing granulomatous inflammation with vasculitis.

After treating the infection, she was treated with oral prednisolone 30 mg once daily for two weeks, which was then tapered. Follow-up chest radiography after four months of treatment showed resolution of lesions (Figure 1, panel d).

Patient B

**Figure 2:**
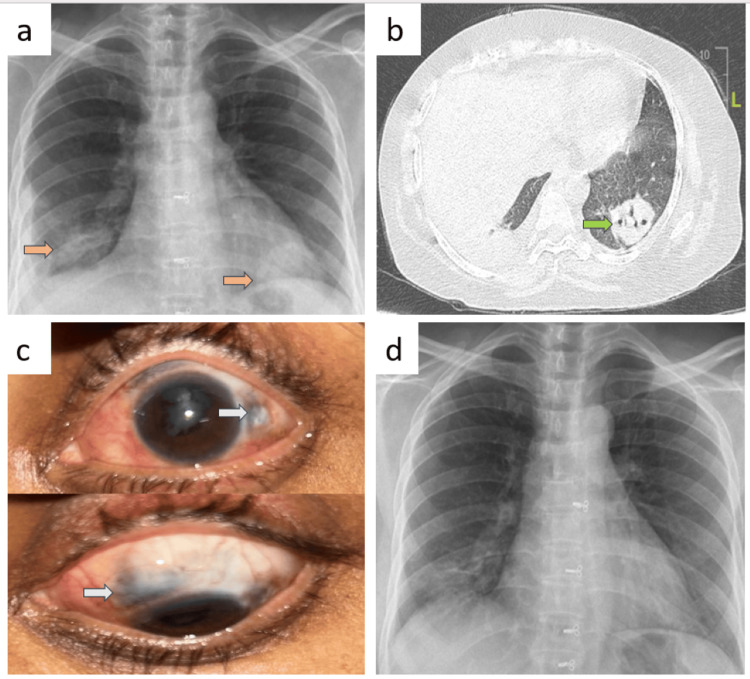
Radiology and ophthalmology images of patient B before and after treatment. (a) Chest X-ray showed a non-homogenous opacity in the right lower zone with costophrenic angle blunting and left lower zone rounded opacity marked by brown arrows. (b) CT showed cavitating consolidation in the left lower lobe marked by a green arrow. (c) The left eye had scleritis and scleral necrosis marked by white arrows. (d) Follow-up chest X-ray showed near-complete resolution.

A 47-year-old housewife reported experiencing redness and watering of her eyes for the past three months and dry cough for the past two months. Her vital signs were stable. Laboratory results showed a random blood sugar level of 170 mg/dL, a white blood cell count of 9,730 cells/mm³, and an elevated platelet count of 579,000 cells/mm³. Urine analysis indicated microscopic hematuria and the presence of (red blood cell) RBC casts. Renal function tests revealed elevated urea at 58 mg/dL, creatinine at 1.7 mg/dL, and a urine protein-to-creatinine ratio (PCR) of 1.52. Liver function tests were normal. Chest X-ray showed a non-homogenous opacity in the right lower zone with costophrenic angle blunting (Figure 2, panel a), while further CT revealed consolidation in the right lower lobe with pleural effusion and cavitating consolidation in the left lower lobe (Figure 2, panel b). Additionally, the patient developed bilateral eye scleritis and scleral necrosis for which she received moxifloxacin and dexamethasone eye drops (Figure 2, panel c). Notably, PR3 c-ANCA level was significantly elevated at over 200 RU/mL confirming the diagnosis of GPA.

BAL bacterial culture grew normal upper respiratory tract flora. The kidney biopsy revealed an ANCA-associated crescentic glomerulonephritis pattern of injury, with late crescentic and necrotizing glomerulonephritis. Due to prominent interstitial histiocytic infiltration and the presence of Langhans-type giant cells, MTB culture and Cartridge Based Nucleic Acid Amplification Test (CBNAAT) were advised to rule out coexisting MTB infection in the kidney.

She was treated with an initial course of intravenous methylprednisolone 1 g once daily, followed by 500 mg daily for the next two days. This was followed by oral prednisolone 40 mg once daily for two weeks, which was then tapered. However, during follow-up, mycobacterial growth indicator tube (MGIT) liquid culture of BAL sample grew *Mycobacterium tuberculosis *after 27 days of incubation, and the patient was initiated on concurrent antituberculosis therapy (ATT). Six months after initiating steroids and ATT, a follow-up chest X-ray showed near-complete resolution (Figure 2, panel d). 

Patient C

**Figure 3:**
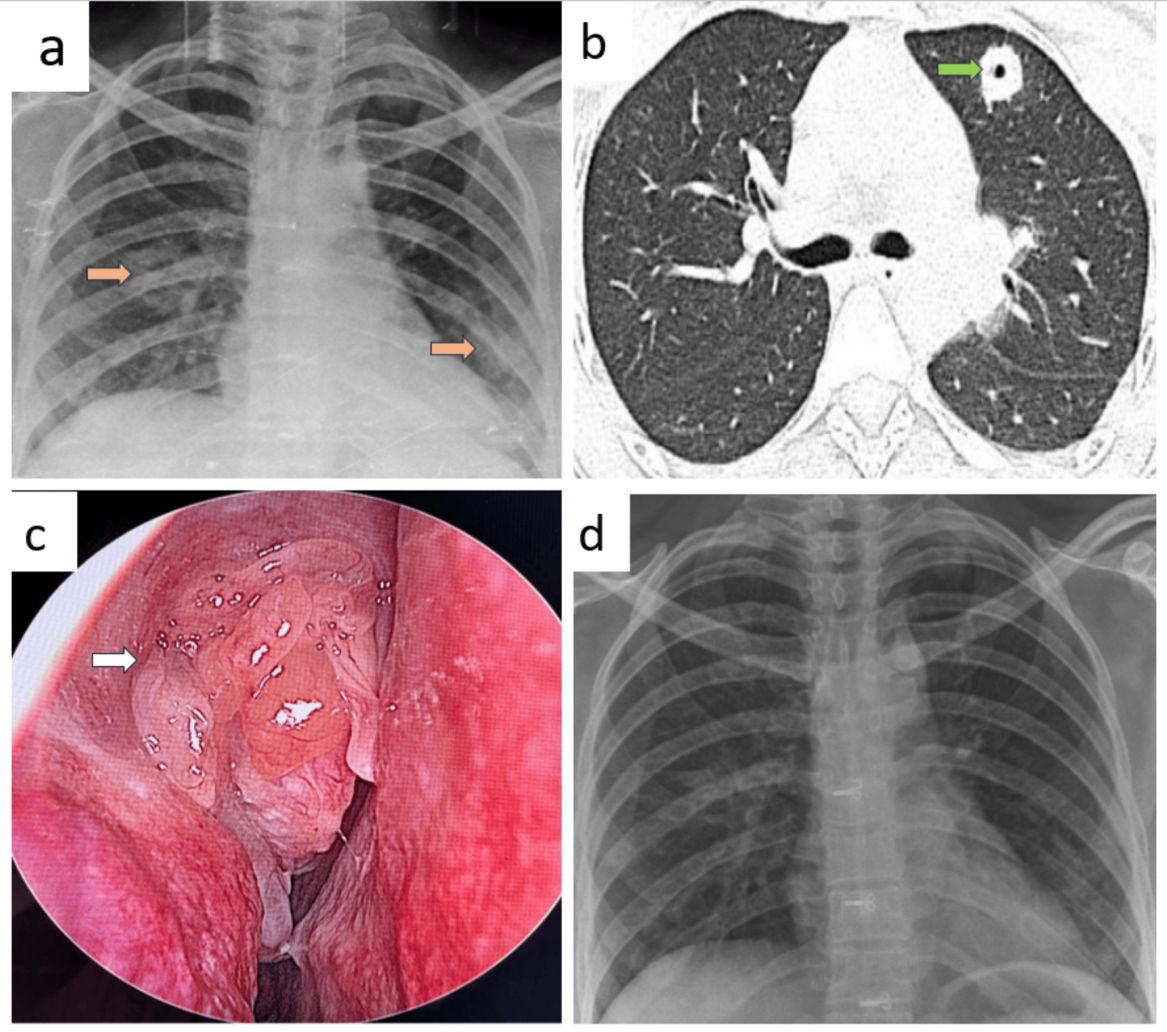
Radiology and anterior rhinoscopy images of patient C before and after treatment. (a) Chest X-ray imaging showed rounded non-homogenous opacities in the right upper and left lower lung zones marked by brown arrows. (b) CT thorax showed left upper cavitating nodule marked by a green arrow. (c) Anterior rhinoscopy examination showed right nasal polyposis marked by a white arrow. (d) Follow-up X-ray showed near complete resolution after four months.

A 44-year-old female presented with a dry cough persisting for 20 days, accompanied by significant weight loss and loss of appetite. Her vital signs were stable. Laboratory investigations revealed a random blood sugar level of 112 mg/dL, WBC count of 13,310 cells/mm³, and a platelet count of 302,000 cells/mm³. Routine urine analysis, renal function test (RFT), and LFT were all normal. Chest X-ray imaging showed rounded non-homogenous opacities in the right upper and left lower lung zones (Figure 3, panel a). Further CT identified bilateral multilobar lung consolidation and cavitating nodules (Figure 3, panel b). She had right nasal polyposis (Figure 3, panel c). The PR3 c-ANCA level was elevated at 92.6 RU/mL, confirming the diagnosis of GPA.

HPE of the nasal polyp and CT-guided lung biopsies revealed features of necrotizing granulomatous inflammation with vasculitis. The patient also had right ASOM caused by methicillin-resistant coagulase-negative staphylococci*,* which was treated with gentamycin ear drops. BAL bacterial culture grew *Burkholderia cepacia*, which was treated with meropenem before initiating steroids. BAL investigations for MTB and fungal infections were negative.

She was treated with oral prednisolone 45 mg once daily for two weeks, which was then tapered, along with methotrexate 10 mg weekly on Fridays and folate 5 mg weekly on Saturdays. Follow-up X-ray showed near-complete resolution after four months (Figure 3, panel d).

Patient D

A 45-year-old male teacher, presented with hemoptysis persisting for 45 days and significant loss of weight and appetite over the past month. Upon clinical examination, his vital signs were stable. Laboratory investigations revealed an RBS of 121 mg/dL, WBC count of 11,500 cells/mm³, and platelet count of 450,000 cells/mm³. Renal function tests and liver function tests were within normal limits. Urine analysis was normal.

**Figure 4:**
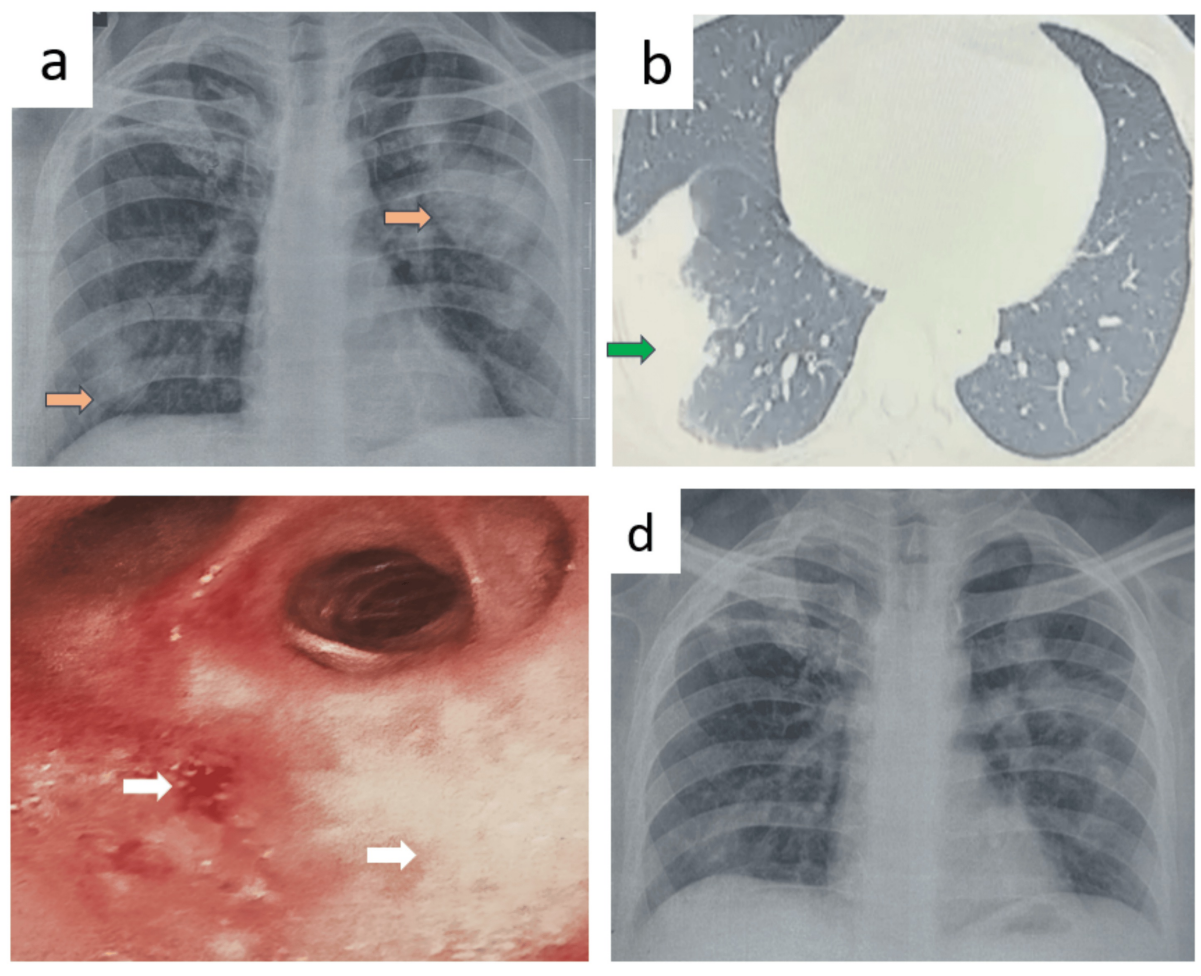
Radiology and bronchoscopy images of patient D before and after treatment. (a) Chest X-ray revealed non-homogenous opacities in the right lower and left upper lung zones marked by brown arrows. (b) CT thorax showed consolidation in the right lower lobe marked by a green arrow. (c) Bronchoscopy showed mucosal ulcerations with whitish membranes and erythema with bleeding spots in the right intermediate bronchus marked by white arrows. (d) After a month, a follow-up chest X-ray demonstrated near-complete resolution of the lung opacities.

Chest X-ray revealed non-homogenous opacities in the right lower and left upper lung zones (Figure 4, panel a). CT thorax showed consolidation in the left upper and right lower lobes (Figure 4, panel b). BAL culture isolated *Pseudomonas aeruginosa*, sensitive to ceftazidime. TB and fungal cultures were negative. The PR3 c-ANCA level was significantly elevated at 121 RU/mL. Bronchoscopy showed mucosal ulcerations with whitish membranes and erythema with bleeding spots in the right intermediate bronchus (Figure 4, panel c). Histopathological examination of an endobronchial mucosal biopsy showed necrotizing granulomatous inflammation with vasculitis.

The patient was administered prednisolone 70 mg daily for two weeks, followed by a tapering regimen, and tablet cyclophosphamide 150 mg daily. After a month, a follow-up chest X-ray demonstrated near-complete resolution of the lung opacities (Figure 4, panel d).

Patient E

**Figure 5:**
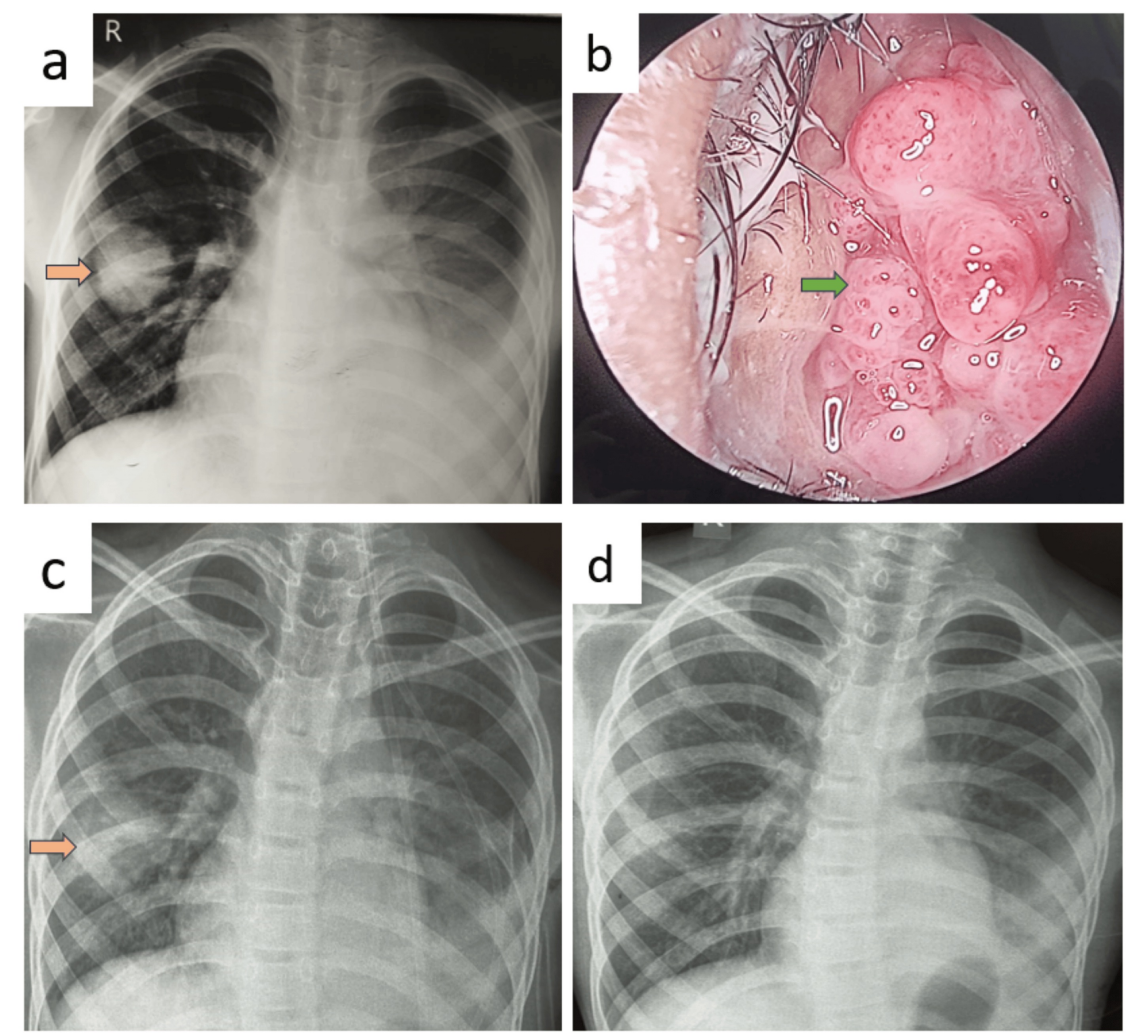
Chest X-ray and anterior rhinoscopy images of patient E before and after treatment. (a) Chest X-ray findings included well-defined rounded opacity in the right mid-lung zone (marked by a brown arrow) and left-sided pleural effusion. (b) Anterior rhinoscopy revealed left nasal polyposis marked by a green arrow. (c) Chest X-ray after a month showed a decrease in the density of the lesion marked by a brown arrow. (d) Three months later, chest X-ray showed near complete resolution of bilateral lesion.

A 20-year-old male mason, complained of epistaxis and nasal stuffiness for four months, accompanied by a dry cough lasting two months. His vital signs remained stable throughout the clinical evaluation. Laboratory tests showed an RBS of 132 mg/dL, WBC count of 8,500 cells/mm³, and platelet count of 350,000 cells/mm³. Both renal and liver function tests were normal, and urine analysis did not indicate any abnormalities. Chest X-ray findings included well-defined rounded opacity in the right mid-lung zone and left-sided pleural effusion (Figure 5, panel a). Examination revealed left nasal polyps (Figure 5, panel b).

The patient’s PR3 c-ANCA level was markedly positive at 180 RU/mL. BAL culture yielded *Klebsiella pneumoniae*, which was sensitive to meropenem. MTB and fungal cultures were negative. Nasal polyp biopsy on HPE demonstrated necrotizing granulomatous inflammation with vasculitis.

The therapeutic regimen included tablet prednisolone 50 mg daily for two weeks, followed by a tapering schedule. Tablet methotrexate 12.5 mg/weekly was administered for remission induction. Chest X-rays after a month (Figure 5, panel c) and three months later showed gradual resolution of the right-sided lesion and left pleural effusion (Figure 5, panel d).

Patient F

A 36-year-old female construction worker, presented with ear discharge for a month fever, and a dry cough persisting for 15 days, along with hematuria over the past 10 days. Clinical examination showed stable vital signs. Laboratory results indicated an RBS of 78 mg/dL, a WBC count of 16,010 cells/mm³, and a platelet count of 250,000 cells/mm³. Urine analysis revealed gross hematuria with RBC casts. Renal function tests showed elevated urea (65 mg/dL) and creatinine (2.5 mg/dL), with a urine PCR of 2.61. Liver function tests were normal.

**Figure 6:**
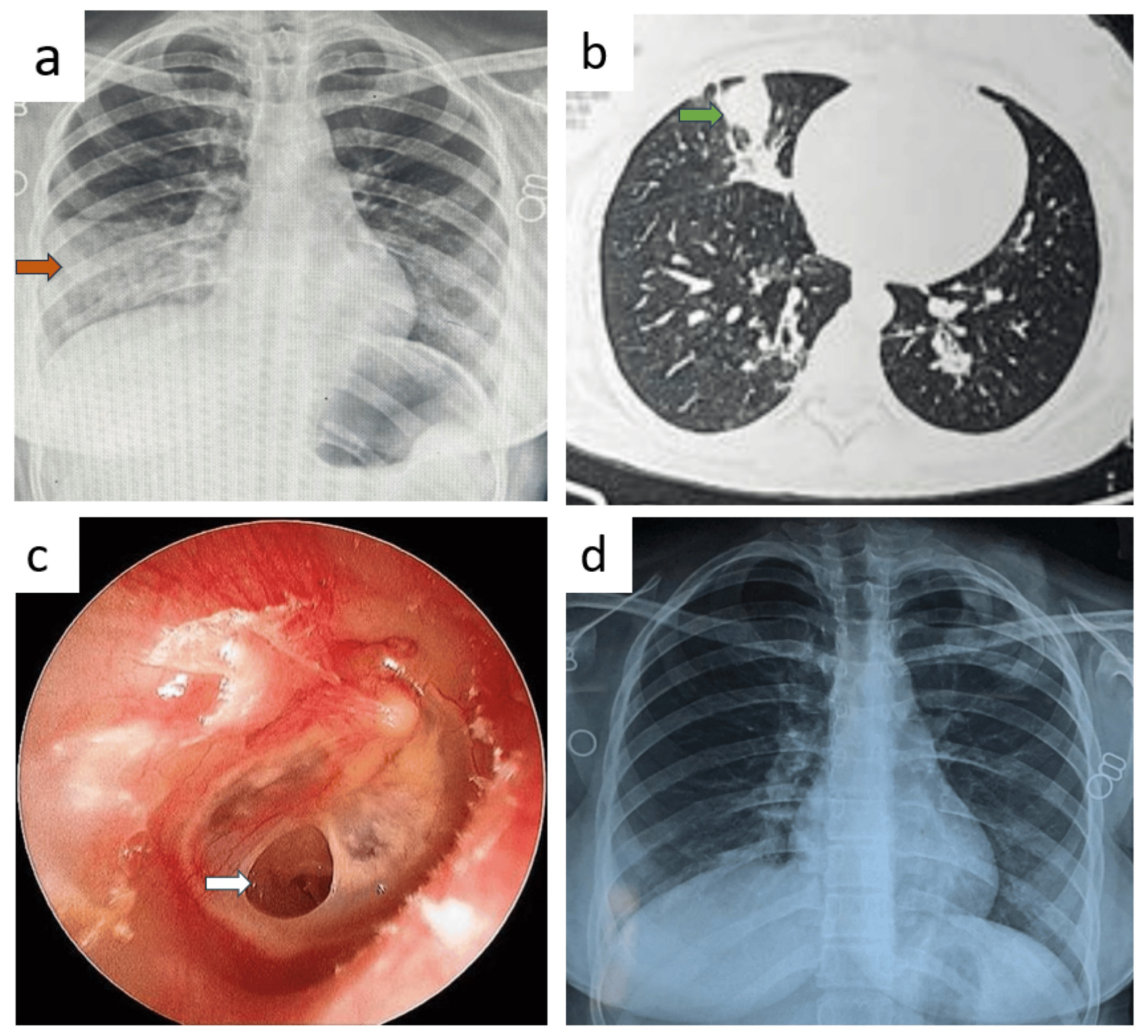
Radiology and otoscopy images of patient F before and after treatment. (a) Imaging studies demonstrated a non-homogenous opacity in the right lower lung zone on chest X-ray marked by a brown arrow. (b) CT showed right middle lobe consolidation marked by a green arrow. (c) Otoscopic image of the tympanic membrane of right ear showing perforation in the anteroinferior quadrant with smooth edges suggestive of chronic otitis media marked by a white arrow. (d) After a treatment period of three months, follow-up imaging showed complete resolution of the opacities.

Imaging studies demonstrated a non-homogenous opacity in the right lower lung zone on chest X-ray (Figure 6, panel a), with consolidation noted in the right middle lobe on CT (Figure 6, panel b). She had right ear chronic suppurative otitis media (CSOM) (Figure 6, panel c). Pus swab culture grew *Staphylococcus aureus*. The nose and eye examinations were unremarkable. The PR3 c-ANCA level was elevated at 232.8 RU/mL. A kidney biopsy on HPE showed ANCA-associated crescentic and necrotizing glomerulonephritis.

The patient was treated with intravenous methylprednisolone 1 g daily for three days, followed by tablet prednisolone 35 mg daily for two weeks. BAL culture grew *Pseudomonas aeruginosa* which was sensitive to ceftazidime and hence treated with the same. MTB and fungal tests were negative. Injection rituximab was given for remission induction and maintenance. After a treatment period of three months, follow-up imaging showed complete resolution of the opacities (Figure 6, panel d).

## Discussion

Case presentations and diagnostic challenges

The cases presented illustrate the diverse and complex nature of GPA, complicated by concurrent infections. There were no similar studies in the literature to compare with this research. Common manifestations include sinonasal lesions, lung nodules, and pauci-immune glomerulonephritis [5]. GPA is characterized by extravascular necrotizing granulomatous inflammation that typically affects the upper and lower respiratory tracts, in addition to vasculitis. Granulomatous lesions in the lung manifest as bilateral nodules and masses, which can be cavitated. Our patients displayed typical signs of GPA, such as a protracted cough, nasal polyps, otitis media, cavitary consolidation in CT, and fibrinoid necrosis in HPE that were in line with vasculitis and granulomatous inflammation [6]. Significantly high PR3 c-ANCA levels in the laboratory results confirmed the diagnosis [7].

In this case series, various concurrent infections required cautious diagnostic assessments and treatment plans. The absence of mucoid or mucopurulent expectoration, fever, and leukocytosis was noteworthy in this case series. This showed that the majority of the patients did not exhibit the typical symptoms, signs, or laboratory findings associated with pneumonia. Prior to the commencement of immunosuppressive treatment, five out of six patients (except patient B) required targeted antibiotic therapy with piperacillin plus tazobactam, meropenem, and ceftazidime due to the presence of *Escherichia coli, Burkholderia cepacia, Pseudomonas aeruginosa, *and *Klebsiella pneumoniae* in their BAL bacterial cultures. *Staphylococcus aureus* has received attention due to reports of increased nasal carriage in relapsing patients with GPA and experimental results linking a plasmid-encoded 6-phosphogluconate dehydrogenase gene from some *S. aureus* strains to myeloperoxidase-ANCA-associated vasculitis [8,9].

*Mycobacterium tuberculosis* was identified in the BAL liquid culture of patient B. Similar cases were found in the literature showing co-existent fungal and mycobacterial infections in patients of GPA [10-12]. Southeast Asia accounts for 48% of the global MTB burden, with extrapulmonary MTB accounting for 17% of cases, according to the World Health Organization Global Tuberculosis Report 2023 [13]. Given that MTB and GPA have similar clinical and radiological characteristics, any attempt to distinguish between both diseases can be difficult, especially in a high TB-burden country like India [14,15]. Classical TB lesions are characterized by the presence of Langhans giant cells and a well-formed spherical granuloma with central caseous necrosis [16,17]. Nonetheless, reports of caseating and non-caseating granulomas exist [17]. The hallmark of GPA, on the other hand, is necrotizing vasculitis of the medium- to small-sized arteries and veins.

Surprisingly, the kidney biopsy of patient B had features of both MTB infection and GPA. This was one of the unique scenarios encountered which had posed a greater diagnostic and therapeutic dilemma. This mandated a multidisciplinary approach involving pulmonologists, microbiologists, pathologists, immunologists, and nephrologists. A shared decision was made to defer maintenance therapy with methotrexate or cyclophosphamide or rituximab due to co-existing tuberculosis, and to administer antituberculosis treatment in addition to corticosteroids. Even without additional immunosuppressants, she had a very good response to ATT and prednisolone. Patients A and C had ASOM whereas patient F had CSOM for which appropriate antibiotic ear drops were administered. They had ear pain and ear discharge but had no deafness. Both upper and lower respiratory infections were treated before starting steroids, with the exception that patients B and F had to receive pulse steroids since they had life-threatening diseases.

GPA patients have been noted to get a variety of infections during the course of their illness when their immunity is dampened by immunosuppressants. However, this case series revealed that the patients had concurrent respiratory illnesses at the time of diagnosis. To add to the mystery, none of them had diabetes mellitus or HIV upon diagnosis. This begs the question of whether the disease pathogenetically has the potential to weaken immunity at the site of involvement, such as the lungs, kidneys, ears, etc., making them more susceptible to infection.

Treatment strategies

**Figure 7:**
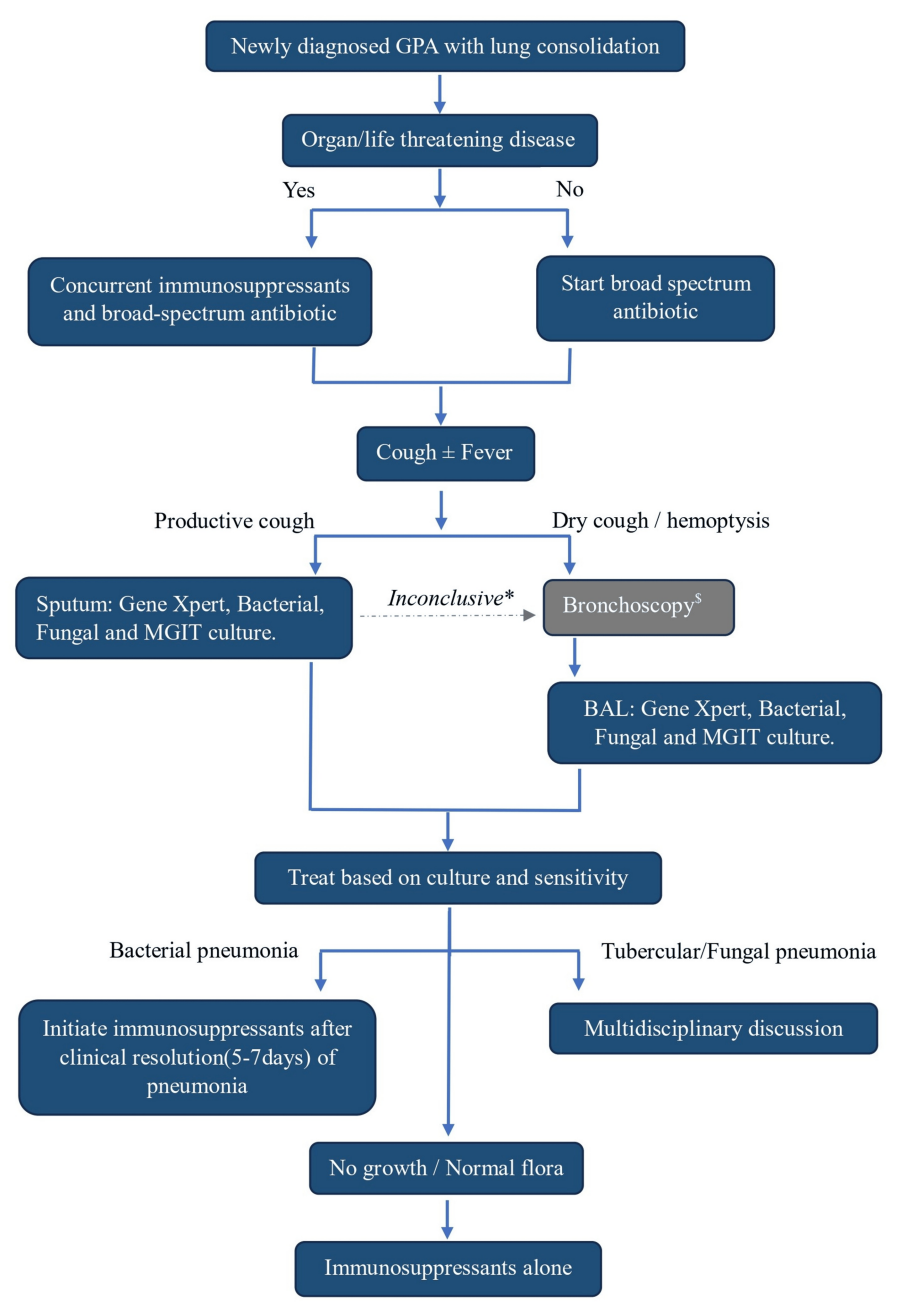
The approach to patients of newly diagnosed GPA with concurrent lung consolidation is depicted in the flow chart. *Inconclusive: if sputum culture grows normal flora or shows no growth, the treating clinician should consider doing bronchoscopy if there is a high index of suspicion of infection, as BAL culture has a higher yield/sensitivity than sputum culture. ^$^Bronchoscopy: dry cough or hemoptysis and cavitary lung consolidation could be symptoms and signs of GPA. Therefore, performing a bronchoscopy to rule out/in infection depends on the treating physician's decision. We were unable to identify a factor that would allow us to suspect infection. This is one of the limitations of the study. GPA: granulomatosis with polyangiitis; BAL: bronchoalveolar lavage; MGIT: mycobacterial growth indicator tube

Each patient's treatment regimen was tailored to address both the infectious and autoimmune components of their condition. In order to eradicate the infection, the right medications had to be used, taking into account culture sensitivity [18,19]. After the infections were successfully treated, immunosuppressive therapy was commenced, which included corticosteroids (prednisolone, methylprednisolone), methotrexate, and, in some cases, cyclophosphamide or rituximab. Controlling the underlying GPA and minimizing the risk of reinfection required careful selection and timing of immunosuppressive drugs. Patients who had organ/life-threatening diseases were given concurrent pulse steroids and broad-spectrum antibiotics. Patients were treated based on the American College of Rheumatology/Vasculitis Foundation Guideline for the Management of Antineutrophil Cytoplasmic Antibody-Associated Vasculitis [5]. At the end of three to four months, all the patients showed clinical and radiological improvement. Studies showed that the addition of co-trimoxazole to standard remission maintenance lowered the likelihood of non-severe relapses in GPA patients, likely due to preventing respiratory tract infections [20,21]. The approach to patients with newly diagnosed GPA with concurrent lung consolidation is depicted in the flow chart below (Figure 7).

Strengths

The study has several notable strengths. Firstly, there were no similar studies in the literature, making this the first study of its kind. Secondly, we provided a simplified flow chart for managing patients with newly diagnosed GPA presenting with lung consolidation. Lastly, the study includes a variety of case presentations along with clinical images, which can help clinicians identify cases with greater ease.

Limitations

Firstly, BAL MGIT culture and CT thorax were not performed in some patients because of unavailability/unaffordability. Secondly, parameters such as erythrocyte sedimentation rate (ESR), C-reactive protein (CRP), neutrophil-lymphocyte ratio (NLR), and procalcitonin were not included in the study. Lastly, symptoms like dry cough, hemoptysis, and cavitary lung consolidation could all be indicative of GPA itself, making bronchoscopy necessary to rule out or confirm infection as determined by the treating physician. Unfortunately, we were unable to identify any factors that would raise suspicion of infection, which represents a major limitation of the study.

In clinical practice, owing to the wide-ranging presentations and treatment strategies of GPA, establishing a GPA registry could be beneficial for creating consensus statement guidelines, archiving records, retrieving information, and supporting future research.

## Conclusions

The present study of the case series highlights the therapeutic complexities of managing patients with granulomatosis with polyangiitis complicated by concurrent infections. Prior to starting immunosuppressive treatment, timely and accurate diagnosis of infections and proper antimicrobial therapy are essential. It also highlighted the need for a multidisciplinary approach involving pulmonologists, rheumatologists, microbiologists, and pathologists in the diagnosis and treatment of GPA with concurrent respiratory infections, emphasizing the importance of individualized treatment plans tailored to the specific clinical scenario. The study questions whether the disease (GPA) could weaken immunity in the affected areas making them more vulnerable to infection (ear: otitis media, lung: pneumonia, and kidney: tuberculosis). Further studies are needed to establish the pathogenesis for the development of infection in a case of GPA (newly diagnosed) and to find the factors that could imply the presence of concurrent infection at various sites (steroid naive).
